# Mitochondrial Fission as a Therapeutic Target for Metabolic Diseases: Insights into Antioxidant Strategies

**DOI:** 10.3390/antiox12061163

**Published:** 2023-05-27

**Authors:** Tianzheng Yu, Li Wang, Lei Zhang, Patricia A. Deuster

**Affiliations:** 1 Consortium for Health and Military Performance, Department of Military and Emergency Medicine, F. Edward Hébert School of Medicine, Uniformed Services University, Bethesda, MD 20814, USA; 2 Henry M. Jackson Foundation for the Advancement of Military Medicine, Inc., Bethesda, MD 20817, USA; 3 Armed Forces Radiobiology Research Institute, Uniformed Services University of the Health Sciences, Bethesda, MD 20814, USA; 4 Department of Pathology, F. Edward Hébert School of Medicine, Uniformed Services University, Bethesda, MD 20814, USA; 5 Center for the Study of Traumatic Stress, Department of Psychiatry, Uniformed Services University of the Health Sciences, Bethesda, MD 20814, USA

**Keywords:** Drp1, reactive oxygen species, exercise, lifestyle interventions, Coenzyme Q_10_, resveratrol, astaxanthin, curcumin, L-citrulline, pharmacological management, mitochondrial integrity

## Abstract

Mitochondrial fission is a crucial process in maintaining metabolic homeostasis in normal physiology and under conditions of stress. Its dysregulation has been associated with several metabolic diseases, including, but not limited to, obesity, type 2 diabetes (T2DM), and cardiovascular diseases. Reactive oxygen species (ROS) serve a vital role in the genesis of these conditions, and mitochondria are both the main sites of ROS production and the primary targets of ROS. In this review, we explore the physiological and pathological roles of mitochondrial fission, its regulation by dynamin-related protein 1 (Drp1), and the interplay between ROS and mitochondria in health and metabolic diseases. We also discuss the potential therapeutic strategies of targeting mitochondrial fission through antioxidant treatments for ROS-induced conditions, including the effects of lifestyle interventions, dietary supplements, and chemicals, such as mitochondrial division inhibitor-1 (Mdivi-1) and other mitochondrial fission inhibitors, as well as certain commonly used drugs for metabolic diseases. This review highlights the importance of understanding the role of mitochondrial fission in health and metabolic diseases, and the potential of targeting mitochondrial fission as a therapeutic approach to protecting against these conditions.

## 1. Introduction

Metabolic diseases are a significant public health concern worldwide due to their high prevalence and impact on individual and societal health [1,2]. Obesity, type 2 diabetes (T2DM), and cardiovascular diseases, among the most common metabolic disorders, are known to increase the risk of other chronic illnesses such as cancer, stroke, and hypertension [3,4,5]. These conditions arise from complex interactions among genetic, environmental, and lifestyle factors, which makes their prevention and treatment challenging [6,7]. The rising global burden of metabolic diseases underscores the need for effective interventions to improve metabolic health and reduce the incidence of related complications. Strategies such as lifestyle modifications, pharmacological interventions, and public health policies are vital in mitigating the impact of these diseases on individuals and populations worldwide. 

One of the underlying mechanisms of metabolic diseases is oxidative stress, which is characterized by an imbalance between the production of reactive oxygen species (ROS) and the antioxidant defense system [8,9,10]. Mitochondria are critical in regulating oxidative stress, as mitochondrial electron transport chain (ETC) complexes are both the main sites of ROS production and the primary targets of ROS, and their dynamic nature plays an essential role in metabolic health [10,11,12,13,14]. Mitochondrial fission is a critical process that regulates the size, number, and function of mitochondria [15,16,17]. In this review, we will explore the physiological and pathological roles of mitochondrial fission and how it can be targeted for antioxidants in metabolic diseases.

## 2. Reactive Oxygen Species: Physiology, Pathology, and Therapeutic Potential 

Reactive oxygen species (ROS) are a group of highly reactive molecules, including superoxide anions (O_2_^•–^), hydrogen peroxide (H_2_O_2_), and hydroxyl radicals (^•^OH). In mammals, they are generated as natural byproducts of cellular metabolism [9,11,13,18]. ROS serve critical roles in cellular signaling and homeostasis, but excessive ROS production can lead to oxidative stress and cellular damage, which contribute to the pathogenesis of several metabolic diseases [9,10,11,19]. ROS can also be induced by exposure to environmental toxins [20], ultraviolet radiation [21], and chemical stressors [22]. Understanding the complex interplay between ROS and cellular homeostasis is essential for developing novel therapeutic strategies for metabolic diseases. 

### 2.1. ROS Generation and Metabolism

**Figure 1:**
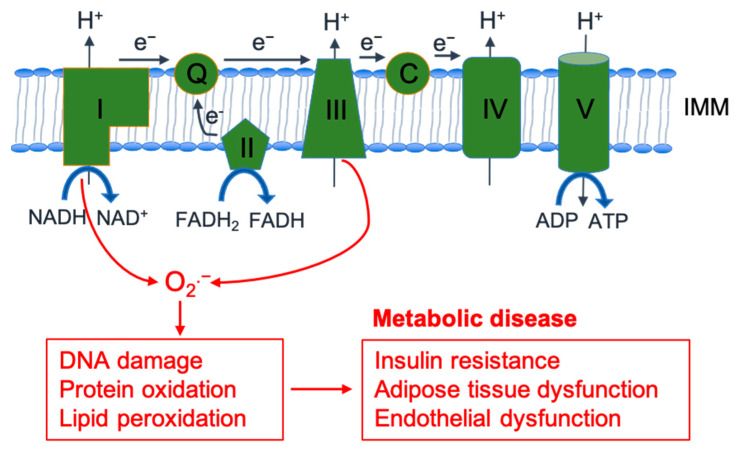
Mitochondrial reactive oxygen species (ROS) production by the electron transport chain and its implications in metabolic disease. Illustration shows ROS production by the electron transport chain (ETC) in the inner mitochondrial membrane (IMM). Electron carriers NADH and FADH2 feed electrons (e^−^) into the ETC at complex I and complex II, respectively. Electrons traverse through the ETC and release energy to pump H^+^ into the intermembrane space, generating a proton gradient and a membrane potential. ATP synthase (Complex V) uses the energy of the proton gradient to generate adenosine triphosphate (ATP) from adenosine diphosphate (ADP) and inorganic phosphate (Pi). Complex I and complex III, where electron leakage can occur, lead to the formation of superoxide radicals (O_2_^•−^) and other ROS. Excessive ROS production can lead to oxidative stress and contribute to the pathogenesis of metabolic diseases. Note: ubiquinone—Q; cytochrome c—C.

ROS are generated by various cellular sources, including mitochondria, peroxisomes, and reduced nicotinamide adenine dinucleotide phosphate (NADPH) oxidases. Mitochondrial ETC complexes are the primary source of cellular ROS, with superoxide anion produced as a byproduct of oxidative phosphorylation (Figure 1) [11,12,13,14]. Peroxisomes are involved in both the production and breakdown of ROS. They contain various types of oxidases, which catalyze the transfer of electrons to molecular oxygen, leading to the production of ROS such as H_2_O_2_ and O_2_^•–^ [23]. NADPH oxidases are membrane-bound enzymes, which produce ROS as defense response to various stimuli, including cytokines, growth factors, microbial pathogens, and metabolic stress [24,25,26]. Of note, NADPH oxidase 4 may locate mitochondria and mediate cross-talk between NADPH oxidase and mitochondria [25,27].

ROS contain unpaired electrons derived from partially reduced molecular oxygen (O_2_) and have a short lifespan. These molecules are continually produced, transformed, and eliminated in various cellular processes. Several defensive mechanisms and mediators of redox signaling in cells help manage ROS at physiological levels. These include: (1) superoxide dismutases (SODs), which convert O_2_^•–^ into less reactive H_2_O_2_ and O_2_ [28]; (2) catalase, which further reduces H_2_O_2_ to water and O_2_ [29]; and (3) glutathione peroxidases, which eliminate H_2_O_2_ through the reducing power derived from glutathione [30]. In addition, other molecules serve a role in redox signaling and act as mediators of defense against ROS. These include peroxiredoxin, thioredoxin (TRX), and the glutathione/glutaredoxin systems [31,32,33]. Together, these defense mechanisms work to regulate the levels of ROS and prevent damage to cells. 

### 2.2. Physiological ROS Signaling

ROS play a role in many physiological processes, including proliferation, differentiation, apoptosis, and immune defense [9]. In particular, ROS have been shown to be important in the regulation of cellular metabolism, including insulin signaling and glucose uptake [34,35]. In physiological conditions, low levels of ROS can activate several transcription factors, including nuclear factor kappa B (NF-κB), activator protein-1 (AP-1), and hypoxia-inducible factor-1α (HIF-1α), which regulate the expression of genes involved in inflammation, angiogenesis, and metabolism [19,36]. 

ROS also serve a role in the regulation of mitochondrial biogenesis through the cyclic adenosine monophosphate (cAMP)/protein kinase A (PKA) signaling pathway [37], and mitophagy, the process by which damaged mitochondria are removed from the cell [38]. In addition, ROS can modulate ion channels and transporters [39], which affect neuronal excitability and synaptic transmission [40], and regulate glucose-dependent insulin secretion by the β-cells [39]. 

### 2.3. The Role of ROS in Metabolic Diseases

ROS can promote oxidative stress, which may lead to deoxyribonucleic acid (DNA) damage, protein oxidation, and lipid peroxidation, and contribute to cellular dysfunction and disease progression (Figure 1) [9]. Excessive ROS production has been linked to the pathogenesis of several metabolic diseases, including obesity, T2DM, and cardiovascular disease [8,12,35]. The dysregulation of ROS production and homeostasis can contribute to the pathogenesis of metabolic diseases through multiple pathways, including insulin resistance, adipose tissue dysfunction (dyslipidemia), and endothelial dysfunction (Figure 1) [34,35,41,42]. The association of oxidative stress with mitochondrial dysfunction and their implications in metabolic diseases are discussed in the next section. 

Insulin resistance, a hallmark of T2DM, is associated with increased ROS production and decreased antioxidant defense. ROS can impair insulin signaling by altering the activity of key enzymes involved in glucose metabolism, such as glycogen synthase and phosphatidylinositol-3-kinase (PI3K), and by inactivating key components of the insulin signaling pathway, including insulin receptor substrate (IRS) and Akt [43,44]. ROS can also activate proinflammatory kinases, such as c-Jun N-terminal kinase (JNK), pro-inflammatory cytokines, such as tumor necrosis factor-alpha (TNF-α) and interleukin-6 (IL-6), and inhibitor of kappa B kinase (IKK), which inhibit insulin signaling by phosphorylating IRS and impairing insulin sensitivity [45,46]. 

Several studies have shown that ROS also play a critical role in the regulation of adipogenesis, the process by which precursor cells differentiate into adipocytes (fat cells) [5,47,48]. ROS can promote adipogenesis by activating the key transcription factor peroxisome proliferator-activated receptor gamma (PPARγ), which regulates the expression of genes involved in lipid metabolism [49]. Increased adipogenesis can lead to the development of obesity, a major risk factor for T2DM and other metabolic diseases. 

Increased ROS production can also cause damage to endothelial cells and impair endothelial function [8]. One of the mechanisms by which ROS contribute to endothelial dysfunction in metabolic disease is through activation of the renin–angiotensin system (RAS). Angiotensin II (Ang II), a key component of the RAS, can increase ROS production and promote inflammation in the endothelium. This leads to impaired nitric oxide (NO)-mediated vasodilation and increased vascular permeability, which contributes to endothelial dysfunction [50]. In addition, Ang II can promote the formation of advanced glycation end-products (AGEs), which are known to contribute to endothelial dysfunction in metabolic disease [51,52].

### 2.4. Therapeutic Potential of ROS

ROS perform critical roles in cellular signaling and homeostasis, but the dysregulation of ROS production can lead to oxidative stress and cellular damage, which may then contribute to the pathogenesis of several metabolic diseases. Understanding the complex interplay between ROS and cellular homeostasis is essential for the development of novel therapeutic strategies for metabolic diseases. 

Several compounds have been studied for their potential therapeutic effects in ROS-related diseases. For example, the antioxidants vitamin C, vitamin E, and resveratrol have been shown to reduce oxidative stress and improve cellular function in various disease models [53,54]. Similarly, inhibitors of NAPDH oxidase (NOX) enzymes, such as apocynin and diphenyleneiodonium (DPI), have been shown to reduce ROS production and improve cellular function in several diseases, including cardiovascular diseases and neurodegenerative diseases [55]. Moreover, compounds that modulate ROS signaling, such as angiotensin-converting enzyme (ACE) inhibitors, have been shown to reduce inflammation and improve cellular function in several diseases [48,52]. The following section will examine the targeting of mitochondrial fission as a potential antioxidant treatment for metabolic diseases.

## 3. Mitochondrial Fission Mechanism and Implications for Metabolic Diseases

Mitochondrial fission is a highly regulated process by which mitochondria tubules divide into smaller fragments. This process is essential for mitochondrial quality control, as it allows for the removal of damaged or dysfunctional mitochondria via mitophagy [56,57,58]. Mitochondrial fission also serves critical roles in energy metabolism, calcium signaling, and apoptosis [13,17,56,57]. The dysregulation of mitochondrial fission has been implicated in the pathogenesis of several metabolic diseases, including obesity, diabetes, and cardiovascular disease [12,13,59]. Understanding the complex interplay between mitochondrial fission and metabolic homeostasis is essential for the development of novel therapeutic strategies for metabolic diseases.

### 3.1. Overview of Mitochondrial Membrane Structure and Dynamics

Mitochondria are double-membrane-bound organelles, with the inner mitochondrial membrane (IMM) containing four large complexes of the ETC, which pump protons across the membrane to generate an electrochemical gradient that is ultimately used by ATP synthase (also called Complex V) to produce ATP through oxidative phosphorylation (Figure 1) [12,13]. The overall morphology of mitochondrial networks is determined by the balance between fusion and fission events (collectively termed mitochondrial dynamics), which maintain their functional and structural integrity [12,13]. 

Mitochondrial fusion is facilitated by the outer mitochondrial membrane (OMM) proteins mitofusin 1 and 2 (MFN1 and 2) and the IMM protein optic atrophy 1 (OPA1) [60,61]. Mitochondrial fusion contributes to the distribution of genetic material and the maintenance of a functional mitochondrial network [62]. The molecular mechanisms of mitochondrial fusion have been reviewed extensively elsewhere [56,60,63,64]. Mitochondrial fission is regulated by the cytosolic dynamin-related protein 1 (Drp1) [15,65,66]. In this review, the authors focus on the mitochondrial fission mechanism and how it can be targeted for antioxidants in metabolic diseases. 

### 3.2. Mitochondrial Fission Mediator Drp1 and Its Regulation

**Figure 2:**
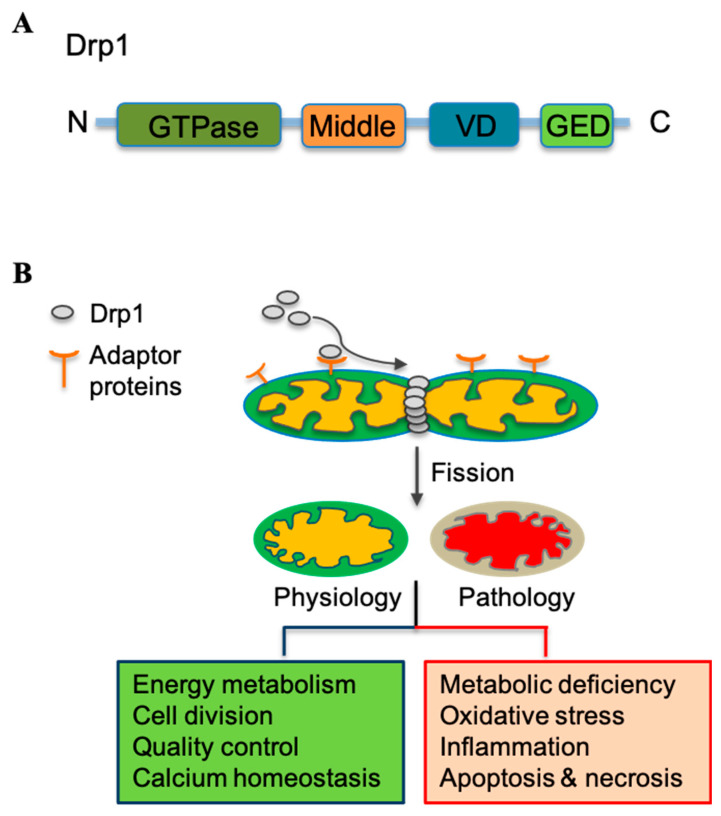
Mitochondrial fission mechanism and its role in physiology and pathology. (**A**) Schematic illustration of dynamin-related protein 1 (Drp1) structure. From the N-terminal to C-terminal, Drp1 is composed of a GTPase domain, middle domain, variable domain (VD), and GTPase effector domain (GED). (**B**) Drp1 is recruited to the outer mitochondrial membrane by the adaptor proteins (i.e., Fis1 and Mff), where Drp1 assembles into a ring-like structure around the mitochondrial tubules and constricts them, leading to their division into two daughter mitochondria. Drp1-mediated fission has broader physiological functions, whereas the dysregulation of Drp1 has been linked to a variety of pathological conditions.

Drp1 is a 78 kDa cytosolic protein that belongs to the dynamin family of large GTPases (Figure 2A). This protein is composed of several domains, each of which has a specific function [12,67]. The N-terminal domain of Drp1 contains a GTPase domain, which is responsible for the GTP-binding and GTPase activity of the protein. The middle domain of Drp1 is the membrane-binding domain that interacts with OMM and is responsible for recruiting Drp1 to the site of fission. The GTPase effector domain (GED) of Drp1 is located at the C-terminus and is involved in the regulation of Drp1 activity. The GED domain interacts with the GTPase domain to regulate the hydrolysis of GTP, which is required for Drp1 function in mitochondrial fission. The variable domain (VD) is located between the middle and GED domains. This domain is not well-understood, but has been proposed to be involved in regulating Drp1 function through interactions with other proteins (Figure 2A).

Upon activation, Drp1 translocates to the OMM and assembles into oligomeric complexes that constrict the membrane to initiate fission (Figure 2B) [15,67,68]. Drp1 is recruited to the OMM by a variety of adaptor proteins, including mitochondrial fission 1 protein (Fis1), mitochondrial fission factor (Mff), and mitochondrial dynamics protein of 49 kDa (MiD49) and of 51 kDa (MiD51) [69,70]. These adaptor proteins contain domains that interact with Drp1 and help to localize it to the mitochondrial surface. 

Ganglioside-induced differentiation-associated protein 1 (GDAP1) may promote the assembly and activation of Drp1 on the OMM [71]. Once recruited to the OMM, Drp1 assembles into higher-order structures that wrap around the mitochondrion, constricting it through a GTP hydrolysis-dependent mechanism until it eventually divides into two daughter mitochondria (Figure 2B) [15,68]. This process requires the coordinated action of other proteins, including the GTPase dynamin-2 and various accessory proteins that facilitate the membrane remodeling events required for fission to occur [66]. 

Drp1 is regulated by several post-translational modifications, including phosphorylation, ubiquitination, SUMOylation, and S-nitrosylation. Drp1 phosphorylation occurs mainly at two serine residues: Ser616 and Ser637. The phosphorylation of Drp1 at Ser616 by cyclin-dependent kinase CDK1 and PKA enhances its GTPase activity, whereas the phosphorylation of Drp1 at Ser637 by calcium/calmodulin-dependent protein kinase I (CaMKI) inhibits its GTPase activity and mitochondrial translocation, resulting in decreased mitochondrial fission. [72,73,74,75]. 

The ubiquitination of Drp1 by an E3 ubiquitin ligase such as Parkin promotes its proteasomal degradation, and hence, inhibits mitochondrial fission [76]. The deubiquitination of Drp1 by ubiquitin-specific protease 30 stabilizes Drp1 and enhances mitochondrial fission [77]. The SUMOylation of Drp1 in the GTPase domain and B domain of Drp1 involves the covalent attachment of a member of the SUMO (small ubiquitin-like modifier) family of proteins to lysine residues to increase its activity, whereas the deSUMOylation of Drp1 by the SUMO-specific peptidases SENP5 and SENP3 has been reported to decrease Drp1 activity and inhibit mitochondrial fission [78]. The S-nitrosylation of Drp1 at cysteine residues by NO inhibits Drp1 activity and mitochondrial fission [79,80].

### 3.3. Pathological Role of Mitochondrial Fission in Metabolic Diseases—The Interplay between Mitochondria and ROS 

**Figure 3:**
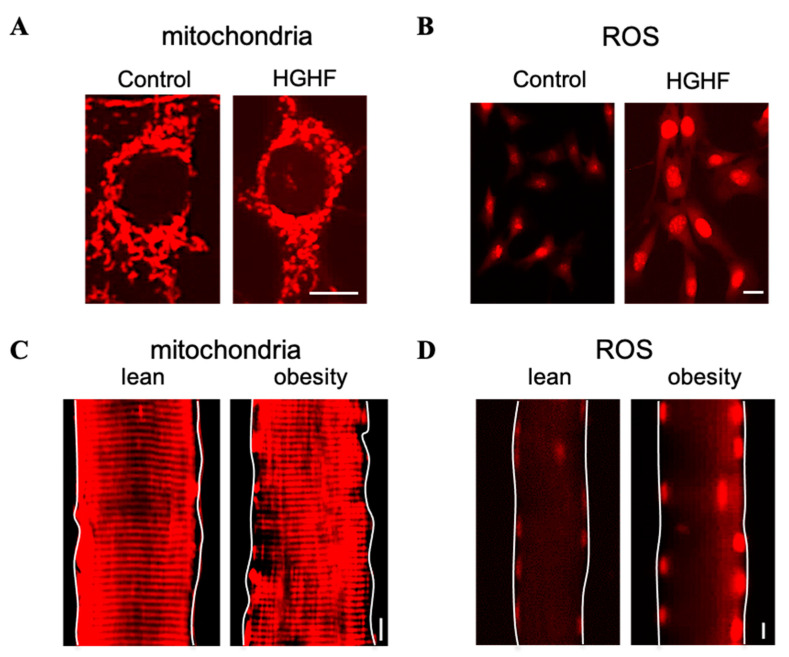
Mitochondrial fragmentation is associated with ROS overproduction in diabetes and obesity. (**A**,**B**) Mouse C2C12 cell line myoblasts maintained in medium containing 5.6 mM glucose, or treated for 2 h with 15 mM glucose (HG) and 0.25 mM palmitate (HF). (**C**,**D**) Flexor digitorum brevis myofibers isolated from lean and obese rats. Mitochondria were labeled with MitoTracker^TM^ Red (**A**,**C**), and ROS levels were detected using the superoxide indicator dihydroethidium (**B**,**D**); scale bar: 10 μm. For methods and quantified results, please refer to our previous publications [40,83].

Mitochondria are dynamic organelles that constantly change their shape and size in response to energy demand and nutrient availability. During exercise or periods of starvation, mitochondria become elongated and more interconnected in skeletal muscle and other tissues, which allows them to be more efficient in producing ATP. This elongation is regulated by Drp1 Ser637 dephosphorylation and a reduction in the fission proteins Drp1 and Fis1, as well as activation of the key mitochondrial biogenesis inductors PGC-1α and upregulation of the levels of the fusion proteins MFN1/2 and OPA1 [81,82,83,84]. Conversely, nutrient excess, such as high glucose/lipid levels, excess dietary fatty acids, or high fructose, can lead to increased mitochondrial ROS production and mitochondrial fragmentation in many cell types, including hepatocytes and skeletal muscle cells (Figure 3) [41,85,86,87,88]. 

The activation of mitochondrial fission is regulated by several proteins, including Drp1 and its activators, such as receptor-interacting protein kinase 1 (RIPK1) and protein kinase Cδ (PKCδ). Under conditions of hyperglycemia and/or hyperlipidemia, Drp1 is phosphorylated by Rho-associated protein kinase 1 (ROCK1) and extracellular signal-regulated kinase 1/2 (ERK1/2), which leads to its translocation to the OMM and the initiation of fission [75,89]. This process is facilitated by an increase in intracellular calcium levels and a decrease in mitochondrial membrane potential, which activate PKCδ and RIPK1, respectively [75,89]. Increased mitochondrial fragmentation has been reported in various tissues in animal and human models of diabetes, obesity, and cardiovascular disease, including liver, muscle, kidney, blood vessel, and adipose tissue [41,90,91,92,93,94].

Mitochondrial fragmentation has been linked to an increase in ROS production (Figure 3), which leads to oxidative stress and cellular damage and contributes to metabolic disease progression (Figure 1) [12,13]. Mitochondria produce ROS as a byproduct of the ETC during oxidative phosphorylation, which is tightly regulated by several factors, including ETC complex activity and mitochondrial membrane potential (Figure 1) [13]. One proposed mechanism is that excessive mitochondrial fragmentation can induce disruption of the IMM and cristae structure, thus causing deformities in ETC complexes, which disrupt electron transfer and proton transport [95,96,97]. As a result, electron and proton leak occur, which leads to decreased ATP production and increased ROS production [12,13]. Additionally, ROS can activate Drp1, a protein responsible for mitochondrial fission, and lead to further mitochondrial fission and ROS production, thus creating a positive feedback loop [98,99]. Finally, the dysregulation of mitochondrial fission can lead to impaired mitophagy and the accumulation of damaged mitochondria, which can then contribute to oxidative stress and inflammation in metabolic diseases [17,56,57,58,64].

Mitochondria are the main target of ROS. They are more susceptible to ROS-induced damage compared to other organelles within cells, and this is due to several factors: First, the ETC, located on the IMM, is a major source of ROS production, which exposes mitochondria to high levels of ROS and makes them more susceptible to damage [9,11,12,13,99]. Second, mitochondria have limited antioxidant defenses compared to other organelles. Although they do contain some antioxidant enzymes (e.g., superoxide dismutase and glutathione peroxidase), these defenses are not as robust as those found in other cellular compartments [38,100]. Third, mitochondria contain their own DNA (mtDNA) that encodes 13 subunits of the ETC complex. mtDNA is more susceptible to oxidative damage than nuclear DNA because it lacks protective histones. As a result, mtDNA damage may lead to mutations and deletions that can impair mitochondrial function and contribute to oxidative stress and metabolic diseases [100,101]. Fourth, the lipids, particularly cardiolipin that is present almost exclusively in the IMM, are susceptible to oxidative damage, which can lead to lipid peroxidation and the disruption of mitochondrial membrane structure and function [100,102,103,104]. Finally, ROS can trigger the mitochondrial permeability transition pore, the release of apoptogenic factors, and the activation of cell death pathways, such as apoptosis, necrosis, or autophagy-mediated cell death [12,17,105].

Several studies have demonstrated the importance of mitochondrial fission in the pathogenesis of metabolic disorders. ROCK1 knockout mice have shown less mitochondrial fragmentation, reduced levels of ROS, and decreased apoptosis in kidney glomeruli under diabetic conditions [89]. Similarly, inhibiting mitochondrial fission by using the dominant-negative fission mutant Drp1-K38A has been shown to reduce ROS levels and oxidative stress, and improve liver function, in diabetic mice [92]. In summary, hyperglycemia and/or high-fat diets promote mitochondrial fission and increase ROS production, which leads to a range of metabolic complications. The mechanism of this process involves the activation of Drp1 by various kinases, intracellular calcium levels, and mitochondrial membrane potential. Numerous recent review articles have emphasized the connections among specific metabolic diseases, mitochondrial fission/fusion, and ROS signaling. We recommend that readers interested in exploring this topic further refer to these articles [106,107,108,109,110,111].

## 4. Target Mitochondrial Fission for Antioxidants in Metabolic Diseases

**Figure 4:**
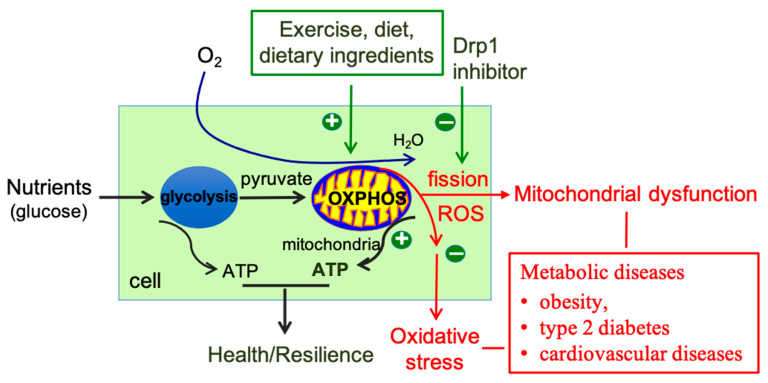
Targeting mitochondrial fission for antioxidants in metabolic diseases. Illustration shows that targeting mitochondrial fission represents a promising strategy for treating metabolic diseases by reducing ROS production and preventing mitochondrial damage. The relationship between ROS and mitochondria in metabolic diseases is complex and bidirectional, with mitochondria being both the primary sites of ROS production and targets of their damaging effects. Approaches such as lifestyle modifications (exercise and diet), dietary supplements, and Drp1 chemical inhibitors have been explored to target mitochondrial fission, and some have shown promising results in reducing ROS production and improving metabolic outcomes. Note: oxygen—O_2_; water—H_2_O; oxidative phosphorylation—oxphos.

As discussed above, Drp1-mediated mitochondrial fission serves a critical role in regulating mitochondrial function and ROS production, and the dysregulation of mitochondrial fission has been linked to the pathogenesis of several metabolic diseases. Lifestyle factors, such as exercise and diet, as well as some dietary supplement ingredients, have been shown to improve mitochondrial function and reduce ROS production by modulating mitochondrial fission. Moreover, chemicals targeting mitochondrial fission, such as mitochondrial division inhibitor-1 (Mdivi-1) and elamipretide (SS-31), have emerged as potential therapeutic agents for metabolic diseases. In this section, we discuss the potential benefits of targeting mitochondrial fission using various approaches, including lifestyle modifications, dietary supplements, and chemicals (Figure 4). We also discuss the interplay between several widely used metabolic disease drugs and mitochondrial dynamics, particularly mitochondrial fission.

### 4.1. Effect of Lifestyle on Mitochondrial Fission and ROS

*Exercise* is a well-established intervention for the prevention and treatment of metabolic diseases. Exercise has beneficial effects on mitochondrial function and fission. Regular exercise increases mitochondrial biogenesis through the activation of the AMPK/ PGC-1α and CaMK/Mitogen-activated protein kinase signaling pathways [112,113], decreases ROS production by upregulating various antioxidant enzymes [114], and enhances mitochondrial quality control (MCQ) mechanisms by activating autophagy [115,116]. Recent studies also show that exercise can modulate mitochondrial fission [117]. High-intensity interval training, for example, has been shown to increase mitochondrial network volume and decrease mitochondrial fragmentation through NOX2 signaling in skeletal muscle [118]. Swimming as a form of exercise can help alleviate endothelial mitochondrial fragmentation by inhibiting Drp1, which contributes to the positive effects of exercise on vascular function and blood pressure in individuals with hypertension [119]. In the nematode *Caenorhabditis elegans*, a single exercise session triggers a process of mitochondrial fragmentation, followed by fusion after a recovery period in the body-wall muscle [120]. In addition, regular daily exercise sessions can delay mitochondrial fragmentation and the decline of physical fitness [120]. Chronic exercise induces mitochondrial fusion, which leads to improved mitochondrial dynamics and insulin sensitivity in adipose tissue [121].

*Various dietary patterns*, such as high-fat diets and sugar-sweetened beverages, can increase mitochondrial fission and impair mitochondrial function, and thereby contribute to the progression of metabolic diseases [12,13]. On the other hand, calorie restriction has been shown to decrease mitochondrial fission and ROS production [122]. Low-fat diets enriched in complex carbohydrates, fiber, and plant-based proteins have been shown to reduce mitochondrial fission and promote mitochondrial fusion, leading to improved energy metabolism and mitochondrial function [123,124]. This is due in part to the fact that carbohydrates stimulate insulin secretion, which activates the PI3K/Akt pathway, and lead to an increase in mitochondrial biogenesis and function [125]. A diet rich in polyunsaturated fatty acids, such as those found in fish, nuts, and seeds, has been associated with improved mitochondrial function and decreased fission in metabolic diseases [126]. In partiular, omega-3 fatty acids may reduce mitochondrial fission by enhancing membrane fluidity and reducing oxidative stress in human skeletal muscle [127].

*Chronic stress* has been shown to increase mitochondrial fission and ROS production, which can contribute to the development of cardiovascular disease, diabetes, and obesity [128]. This may reflect activation of the hypothalamic–pituitary–adrenal axis and sympathetic nervous system. Chronic activation of these pathways leads to the release of stress hormones such as cortisol and adrenaline, which can affect mitochondrial dynamics and function [128,129,130]. For example, chronic exposure to elevated levels of cortisol or dexamethasone has been shown to increase mitochondrial fission and decrease fusion, which may lead to mitochondrial dysfunction [131,132]. Another mechanism involves the activation of inflammatory pathways in response to chronic stress. Inflammation can lead to the production of ROS and oxidative stress, which can, in turn, affect mitochondrial function and dynamics [133]. The inflammatory cytokine TNF-α can increase mitochondrial fission and ROS production [134]. The pro-inflammatory cytokine IL-6 has also been shown to increase mitochondrial fission and decrease fusion, which leads to mitochondrial fragmentation and dysfunction [135,136]. Thus, stress management techniques, such as meditation and yoga, can help to improve mitochondrial integrity and function. and prevent the development of metabolic diseases.

### 4.2. Effect of Dietary Supplements on Mitochondrial Fission and ROS 

Some ingredients found in dietary supplements may have potential as adjunct therapies for metabolic diseases, particularly when combined with lifestyle interventions such as exercise and dietary changes as discussed above. In this section, we consider several ingredients that have been shown to improve mitochondrial function and reduce oxidative stress in metabolic diseases. Of note, further research is needed to fully elucidate the mechanisms by which these ingredients improve mitochondrial integrity and function, as well as to determine the optimal amounts and combinations for mitigating metabolic diseases.

*Coenzyme Q_10_* (CoQ_10_), a key component of the electron transport chain, is required for ATP production. CoQ_10_ exhibits antioxidant properties by providing electrons to unstable molecules such as free radicals, thereby neutralizing them. CoQ_10_ also aids in the regeneration of other antioxidants such as Vitamin E, and has been shown to improve mitochondrial function and reduce oxidative stress in various animal and human models [137,138]. Several recent studies found that CoQ_10_ supplementation can reduce mitochondrial fission and increase mitochondrial fusion, to result in improved mitochondrial function and reduced ROS production [139,140,141]. In a mouse model of obesity and T2DM, CoQ_10_ supplementation improved lipid metabolism and mitigated obesity, possibly through CaMKII-mediated phosphodiesterase-4 inhibition [142]. Clinical studies have shown that CoQ_10_ supplementation can improve insulin resistance, glycemic control, and lipid metabolism in diabetic patients, to potentially reduce the risk of diabetic complications [143]. In obesity, CoQ_10_ supplementation has been shown to improve mitochondrial function and reduce inflammation, which is a hallmark of obesity-induced insulin resistance [144]. These findings suggest that CoQ_10_ may have potential as a therapeutic agent for the management of metabolic diseases.

*Resveratrol* is a polyphenol found in grapes, berries, and other plants. Recent studies have shown that resveratrol has antioxidant properties and can improve mitochondrial function in metabolic diseases. Resveratrol can activate sirtuin 1, a key regulator of mitochondrial biogenesis and function [145,146,147,148]. Resveratrol has been shown to promote mitochondrial fusion and decrease the expression of the fission proteins Drp1 and Fis1, which leads to a decrease in mitochondrial fission [145,146,149]. This decrease in mitochondrial fission results in an improvement in mitochondrial function, including increased ATP production, reduced oxidative stress, and improved insulin sensitivity [147,148,150,151]. Resveratrol also reduces oxidative stress by increasing the activity of antioxidant enzymes [152]. Clinical studies have suggested that resveratrol supplementation may have beneficial effects on metabolic diseases in both animal models and humans [153,154].

*Astaxanthin* is a carotenoid pigment with potent antioxidant and anti-inflammatory properties. Several studies have investigated the effects of astaxanthin on mitochondrial fission and ROS production in the context of metabolic diseases. In mouse skeletal muscle and hypothalamus, astaxanthin improved mitochondrial biogenesis, inhibited mitochondrial fragmentation, and reduced oxidative damage [155,156]. Astaxanthin can activate the mammalian target of rapamycin (mTOR) pathway, which results in elevated mitochondrial fusion and reduced fission [157]. In the blood vessels, astaxanthin prevented Drp1 Ser616 phosphorylation and the excessive generation of mitochondrial ROS [158]. In rats, astaxanthin reduced Drp1 protein expression and delayed the pathogenesis of diabetic nephropathy [159]. The activation of the AMPK pathway by astaxanthin may contribute to the stimulation of mitochondrial biogenesis and the improvement of insulin resistance [160]. In human subjects, astaxanthin exhibits some advantages in enhancing mitochondrial function and halting the progression of metabolic diseases [161,162]. However, more research is needed to fully understand the potential benefits and mechanisms of astaxanthin in metabolic disease.

*Curcumin*, a polyphenol found in turmeric, has been shown to inhibit mitochondrial fission and improve mitochondrial function in obesity and diabetes. Curcumin regulates ROS hormesis, which can serve to inhibit mitochondrial fission and promote fusion/elongation and biogenesis, and improve function, in the skeletal muscle of rodents, possibly through NADPH oxidase-dependent redox signaling [26,163]. Curcumin treatment in mice resulted in upregulation of the expression of the transcriptional coactivator PGC1α protein, which likely restored mitochondrial fusion and improved mitochondrial function [164]. In human neuroblastoma cells, curcumin was found to increase mitochondrial fusion activity, decrease fission machinery, and enhance biogenesis [165]. The results of clinical studies have been reviewed recently, and suggest that curcumin may be beneficial in patients with metabolic diseases [166,167].

*L-citrulline* is a non-essential amino acid found in watermelon and other foods. L-citrulline is a precursor to L-arginine, a key substrate for the production of nitric oxide (NO) in the body. Excessive NO has been shown to stimulate the S-nitrosylation of Drp1 and promote mitochondrial fission [79]. L-NAME, a non-selective NO synthase inhibitor, abolished the S-nitrosylation and phosphorylation of Drp1 at Ser616 in neurons, which led to elongation of the mitochondria [168]. L-citrulline caused an increase in NO levels in skeletal muscle, which resulted in the reduced phosphorylation of Drp1 at Ser616 and increased phosphorylation at Ser637. As a result, mitochondrial fission was inhibited, and oxidative stress and its associated damage were alleviated. [169]. Animal and human studies suggest that L-citrulline may have potential therapeutic benefits in various metabolic diseases by improving mitochondrial function and reducing oxidative stress, due to the ability of L-citrulline to increase NO production [170,171]. Additional research is necessary to confirm whether supplementation with L-citrulline could be an effective therapeutic approach to preventing and treating metabolic disorders.

### 4.3. Drp1 Chemical Inhibitors

Drp1 inhibitors are a type of chemical compound that can block the activity of Drp1 and prevent it from functioning properly. These inhibitors work by either directly targeting the GTPase activity of Drp1 or disrupting the interactions between Drp1 and its binding proteins. Several Drp1 inhibitors have been developed and studied for their potential use in treating a variety of metabolic disorders, including obesity, T2DM, and cardiovascular diseases. 

*Mitochondrial division inhibitor-1* (Mdivi-1) was first identified as a selective inhibitor of Drp1 in a high-throughput screen of small molecules [172]. It inhibits Drp1 GTPase activity and disrupts the assembly of the Drp1 complex, which then leads to reduced mitochondrial fission [172]. Mdivi-1 has been shown to be effective in various animal models of diseases characterized by dysregulated mitochondrial fission. Mdivi-1 treatment improved mitochondrial function and reduced oxidative injury in the skeletal muscle of heat-stressed mice [173]. It reduced adipose tissue inflammation and improved glucose metabolism in obese mice [174]. Mdivi-1 treatment also improved insulin sensitivity and glucose uptake in adipose tissue and skeletal muscle [174,175,176]. In studies on high-fat diet-induced obesity in mice and obese insulin-resistance in humans, Mdivi-1 was found to reduce body weight, ROS production, and insulin resistance. It improved glucose tolerance and decreased fasting blood glucose levels and reduced oxidative stress and inflammation in the liver and skeletal muscle [174,175,176]. Treatment with Mdivi-1 also improved mitochondrial function and reduced oxidative stress in the hearts of diabetic mice, which led to improvements in cardiac function [177]. In addition to its effects on mitochondrial dynamics, Mdivi-1 has been found to have anti-inflammatory and anti-oxidative properties [178,179,180], and it inhibits autophagy [178,181], which may contribute to its therapeutic effects in metabolic diseases.

*P110* is a peptide inhibitor that disrupts the interaction between Drp1 and Fis1, and inhibits Drp1 enzyme activity [182]. By inhibiting Drp1, P110 prevents excessive mitochondrial fragmentation and protects against mitochondrial dysfunction and cell death [182]. P110 and Mdivi-1 have been shown to reduce the translocation of Drp1 to the mitochondria and ROS production, which led to improved mitochondrial and heart function [183,184,185]. P110 reduced oxidative stress in neurons and improved mitochondrial function, motor performance, and survival [186]. This effect may be mediated through TNF-α-induced inflammation, H_2_O_2_-induced oxidation, and/or the RIPK1/RIPK3 pathway [187]. P110 may also abolish the association of p53 with the mitochondria and reduce brain infarction in rats subjected to brain ischemia/reperfusion injury [188]. P110 was able to decrease Drp1 Thr595 phosphorylation and reduce mitochondrial dysfunction [189]. Overall, these studies suggest that Drp1 inhibitors such as P110 have the potential to improve metabolic function and may be useful in the treatment of metabolic diseases [190,191]. 

*Dynasore* is a small molecule that can inhibit Drp1 activity by blocking the activity of dynamin, a protein involved in the formation of membrane vesicles [192]. Dynamin is involved in the regulation of glucose transporter 4 trafficking, which is essential for insulin-mediated glucose uptake [193]. One of the main mechanisms by which Dynasore may exert its effects on metabolic diseases is through the inhibition of glucose uptake. Glucose uptake is a critical step in the development of metabolic diseases, as increased glucose uptake by cells leads to insulin resistance and impaired glucose homeostasis [194]. Dynasore has been shown to improve insulin resistance and glucose metabolism in human liver cells [195]. The dysregulation of lipid metabolism is a hallmark of metabolic diseases, and the inhibition of endocytosis by Dynasore has been shown to inhibit lipolysis and improve lipid metabolism in adipocytes [196]. In human HeLa cells and macrophages, treatment with Dynasore resulted in a decrease in the levels of intracellular cholesterol and impaired the trafficking of cholesterol from the plasma membrane to the endoplasmic reticulum [197]. In addition, Dynasore directly inhibited the mitochondrial fission protein Drp1 and reduced oxidative stress in cardiomyocytes under ischemic/reperfusion conditions, which led to increased cardiomyocyte survival and improved overall cardiac function [198].

### 4.4. Effect of Metabolic Disease Drugs on Mitochondrial Fission and ROS 

*Metformin*, a commonly prescribed medication for T2DM, has been extensively studied for its effects on mitochondrial function. Metformin has been shown to inhibit mitochondrial fission and promote mitochondrial fusion [199,200]. It exerts its effects by activating AMPK, which, in turn, inhibits Drp1-mediated mitochondrial fission [199,200]. Interestingly, several recent studies have shown that metformin improves mitochondrial respiration by increasing mitochondrial fission through AMPK-Mff signaling [201], or by phosphorylating AMPK/Drp1 Ser616 [202]. Nevertheless, in patients with T2DM, metformin restored mitochondrial fission/fusion balance and improved mitochondrial function [203]. Additionally, metformin has antioxidant properties and can reduce ROS production, leading to decreased oxidative stress [204]. These effects contribute to the potential benefits of metformin in mitigating mitochondrial dysfunction and oxidative damage associated with metabolic diseases. 

*Sodium–Glucose Cotransporter-2 inhibitors (SGLT-2i)* are a class of medications used to lower blood glucose levels in individuals with T2DM. A recent review article has summarized that SGLT-2 inhibitors may have direct effects on mitochondrial function [205]. SGLT-2i treatment has been shown to increase mitochondrial biogenesis and improve mitochondrial function in various tissues, including the heart and kidney [205]. While the exact impact on mitochondrial fission is not yet fully understood, the beneficial effects of SGLT-2 inhibitors on mitochondrial function may directly or indirectly influence mitochondrial fission and fusion proteins [206,207,208,209]. Furthermore, SGLT-2 inhibitors have been reported to reduce oxidative stress and ROS production, potentially through their glucose-lowering and hemodynamic effects, which can contribute to their overall metabolic benefits [210]. 

*Statins* are widely used cholesterol-lowering drugs that inhibit the enzyme HMG-CoA reductase, a key enzyme in cholesterol synthesis [211]. Statins have been reported to affect mitochondrial function [212,213], but their effects on mitochondrial fission are not well-defined. Some studies have reported that statin treatment can reduce the expression and activity of the mitochondrial fission proteins Drp1 and Fis1 [214,215,216]. However, the results from different studies are conflicting, with some reporting an increase in fission-related proteins/mitochondrial dysfunction and others showing no significant effect of statin treatment [213,217,218,219]. It is likely that the effects of statins on mitochondrial dynamics vary depending on the specific statin, its dosage, the duration of treatment, and the experimental model or tissue studied. Further research is needed to elucidate the precise mechanisms and determine the consistent effects of statins on mitochondrial fission and fusion.

*ATP-citrate lyase (ACLY) inhibitors* are emerging as potential therapeutics for metabolic diseases and cancer, targeting key enzymes involved in de novo lipogenesis [220]. ACLY plays a role in providing acetyl-CoA, which is known to modulate the post-translational modifications of proteins, including lysine acetylation [220,221]. The acetylation of Drp1 has been shown to increase its activity and mitochondrial localization, thereby activating mitochondrial fission [222]. By inhibiting ACLY and reducing the availability of acetyl-CoA, mitochondrial fission could be indirectly affected. However, the specific consequences would depend on the context and the overall metabolic state of the cell. Further research is needed to fully understand the relationship between ACLY inhibition and mitochondrial fission and to elucidate the molecular mechanisms involved.

## 5. Conclusions and Perspectives

In conclusion, targeting mitochondrial fission represents a promising strategy for mitigating mitochondrial dysfunction and oxidative stress in metabolic diseases. The balance between ROS production and scavenging is tightly regulated, and mitochondrial fission serves a critical role in this process. By inhibiting mitochondrial fission or enhancing antioxidant capacity, it may be possible to restore mitochondrial homeostasis and improve metabolic health (Figure 4). 

However, there are limitations and challenges to consider in targeting mitochondrial fission for metabolic disease treatment. For example, the role of mitochondrial fission in metabolic diseases is complex and involves multiple factors, and the optimal duration and intensity of mitochondrial fission inhibition are not yet clear. In addition, the effects of long-term mitochondrial fission inhibition on other physiological processes, such as cellular respiration and autophagy, need to be further investigated. Furthermore, effective and safe mitochondrial fission inhibitors with clinical relevance are lacking. 

While mitochondrial fission has been extensively discussed in this review, it is important to acknowledge the role of mitochondrial fusion in maintaining mitochondrial function and metabolic health. Studies have shown that abnormalities in mitochondrial fusion can also lead to metabolic dysfunction and contribute to the development of metabolic diseases such as obesity, insulin resistance, and T2DM [12,106,223]. In particular, impaired mitochondrial fusion has been associated with reduced oxidative capacity and impaired glucose uptake in skeletal muscle and adipose tissue, which are important tissues for whole-body glucose homeostasis [106,224].

Therefore, future studies should focus on identifying the specific mechanisms underlying mitochondrial fission and ROS production in metabolic diseases, as well as on developing novel and effective mitochondrial fission inhibitors with minimal side effects. Moreover, it is important to explore the potential synergistic effects of targeting mitochondrial fission with other antioxidant therapies, such as lifestyle modifications and dietary supplements. A better understanding of the complex relationships and interactions among mitochondrial fission/fusion, ROS, and metabolic diseases will ultimately lead to the development of new and effective therapeutic strategies for these conditions.
